# An Electrohydrodynamic Phase-Field Model for Contact Angle Hysteresis in Electrowetting Pixels: Decoupling Physical Pinning and Charge Trapping

**DOI:** 10.3390/mi17040480

**Published:** 2026-04-15

**Authors:** Qingsong Lu, Li Wang, Feng Li, Yanjun Yang, Qifu Liu, Xinying Wang, Feng Chi, Liming Liu, Zichuan Yi

**Affiliations:** 1 School of Electronic Information, University of Electronic Science and Technology of China, Zhongshan Institute, Zhongshan 528402, China; 2024024402@m.scnu.edu.cn (Q.L.); 202322310333@std.uestc.edu.cn (F.L.); 2024024416@m.scnu.edu.cn (Y.Y.); l13137360827@163.com (Q.L.); xinying_wang@163.com (X.W.); chifeng@semi.ac.cn (F.C.); liulmxps@126.com (L.L.); 2 Guangdong Provincial Key Laboratory of Optical Information Materials and Technology, South China Academy of Advanced Optoelectronics, South China Normal University, Guangzhou 510006, China; 3 School of Information Engineering, Zhongshan Polytechnic, Zhongshan 528400, China; wangli@zspt.edu.cn

**Keywords:** electrowetting, contact angle hysteresis, electrohydrodynamics, phase-field method, molecular kinetic theory, charge trapping

## Abstract

Precise manipulation of two-phase flow in micro-confined electrowetting pixels is limited by contact angle hysteresis (CAH). To elucidate this non-equilibrium process, we establish a high-fidelity electrohydrodynamic (EHD) phase-field simulation framework. The model rigorously couples Navier–Stokes equations with molecular kinetic theory (MKT) to characterize energy dissipation at the three-phase contact line (TCL) and further integrates charge transport kinetics. Numerical results reveal CAH is driven by physical pinning and interfacial charge trapping, with the latter dominating interfacial retreat and causing significant residual displacement. Furthermore, analysis shows alternating current (AC) waveforms mitigate charge accumulation and promote depinning via micro-oscillations, minimizing the hysteresis loop compared to direct current (DC) waveforms. Additionally, an overdrive strategy utilizing a suprathreshold Maxwell stress pulse rapidly overcomes static friction. This strategy significantly improves transient dynamics, substantially reducing the time to reach 90% of the steady-state target from 19.6 ms (under standard DC waveform driving) to 7.4 ms. This work provides a comprehensive theoretical basis and design criteria for optimizing active driving strategies in optofluidic and digital microfluidic systems.

## 1. Introduction

From displays to micro-interface dynamics, electrowetting has emerged as a ubiquitous mechanism for the precise manipulation of conductive fluids at the microscale, with extensive applications in digital microfluidics, tunable liquid lenses, and reflective displays [1,2]. Within reflective display pixels, modulating the interfacial tension via an external electric field enables the reversible spreading and retraction of droplets in confined geometries [3,4]. The rapid switching of these oil–water interfaces governs optical transmission, making the fundamental fluid dynamics of these systems a complex EHD challenge rather than a mere engineering concern [5]. Crucially, the precise control of the fluid interface is strictly limited by CAH, a phenomenon that manifests as the asymmetric response of the contact line during the advancing and receding phases [6].

While CAH is entirely absent in ideal static scenarios, where the wetting state is uniquely determined by the Young–Lippmann equation balancing capillary and electrostatic forces, practical electrowetting pixels operate in a dynamic, non-equilibrium regime [7,8]. Here, the TCL motion is impeded by multiple dissipation mechanisms. The origins of CAH in these systems are tripartite. First, from a hydrodynamic perspective, the viscous dissipation within the bulk fluid and the singular stress near the moving contact line create resistance to motion, a behavior often described by the MKT [9]. Second, physical pinning arises from surface heterogeneity and roughness at the dielectric interface, requiring the system to overcome local energy barriers to initiate depinning [10]. Third, and most critically, under high-field driving, charge trapping occurs at the fluid–solid interface. The injection of charge carriers into the dielectric layer establishes a remnant internal electric field. This field induces an electrostatic screening effect, effectively reducing the Maxwell stress acting on the interface and exacerbating the hysteresis loop from an electrical perspective [11].

Mitigating these hysteresis effects necessitates the optimization of driving waveforms—such as employing AC waveforms or overdrive pulses—to facilitate contact line depinning [12,13,14,15]. However, this waveform optimization currently relies heavily on empirical trial-and-error due to the inadequacy of static theories [16,17]. Classical approaches rooted in the Young–Lippmann equation provide foundational insights [18,19], and recent numerical studies have extensively investigated either interfacial topology or charging kinetics in isolation [20,21]. Despite these advances, a unified multiphysics framework remains to be fully established. Specifically, challenges remain in simultaneously coupling the topological evolution of the two-phase interface, the microscopic energy dissipation at the contact line, and the transient charge transport within the dielectric layer.

To address the aforementioned issues, a high-fidelity multiphysics simulation framework is established in this paper to elucidate the intrinsic mechanisms of CAH in micro-confined electrowetting pixels. First, the diffuse-interface dynamics are captured to ensure mass conservation and accurate stress continuity across the two-phase boundary. Second, the MKT model is explicitly coupled to the wetting boundary conditions to quantify the physical pinning strength. Finally, a charge-trapping kinetic module is integrated to establish a real-time feedback loop between the trapped charge density and the effective electrowetting number. By systematically investigating the transient response under various driving strategies, this framework aims to decouple the competing dissipation mechanisms and provide physical criteria for optimizing the dynamic wetting stability of optofluidic systems.

## 2. Theory

**Figure 1:**
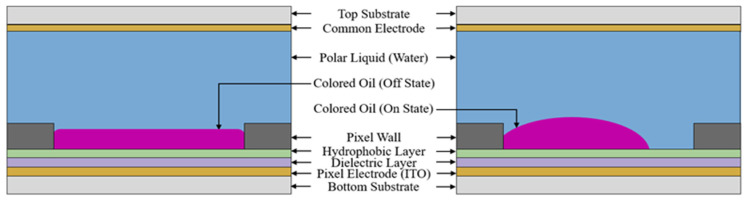
The structure diagram of an electrowetting pixel.

Electrowetting pixels exploit the competition between the electrowetting force and interfacial tension to regulate the contraction and spreading of the colored oil, thereby achieving pixel switching and grayscale modulation [22,23]. A pixel is primarily composed of a multilayer stack, including a bottom substrate, electrodes, a dielectric layer, a hydrophobic layer, a pixel wall, colored oil, polar liquid, and a top plate [24], as shown in Figure 1. As the electric field intensity increases, the initial interfacial tension balance is disrupted. The enhanced wettability of the polar liquid allows it to displace the oil and contact the hydrophobic dielectric surface, driving the oil to contract and revealing the underlying substrate [25,26]. The static equilibrium state and the quantitative relationship between the contact angle and the applied voltage are fundamentally governed by the Young–Lippmann equation [27]. The resulting optical state is intrinsically linked to the degree of oil contraction, which is quantified by the aperture ratio (AR) of the pixel.

While the Young–Lippmann equation describes static equilibrium on ideal surfaces, practical pixels operate in a dynamic, non-equilibrium regime. When driven by dynamic electrical signals, such as stair-step DC waveforms, the pixel exhibits CAH [28,29]. The movement of the TCL at the oil–water–solid interface is impeded by pinning forces. During the voltage rising phase, the TCL requires an electric field force greater than the theoretical value to initiate advancement. Conversely, during the voltage-falling phase, the voltage must drop below the theoretical threshold before the oil can displace the water, resulting in a distinct hysteresis loop where the advancing and receding contact angles do not coincide.

The physical essence of this hysteresis lies in the molecular hydrodynamic resistance encountered during TCL movement [30]. This non-equilibrium process can be described by the MKT, which posits that TCL movement results from the thermally activated jumping of liquid molecules between adsorption sites on the solid surface [31].

Furthermore, the hysteresis loop is not solely a product of physical pinning but is profoundly affected by the charge-trapping phenomenon occurring at the dielectric interface. During pixel operation under the DC waveform, charge carriers can be injected from the polar liquid into the hydrophobic dielectric layer and become sequestered within localized defect states. These trapped charges establish a persistent internal remnant electric field that opposes the externally applied potential. The resulting reduction in the effective voltage effectively shields the TCL from the intended electrostatic pressure, leading to a significant discrepancy in the AR during the voltage ramp-down phase compared to the ramp-up phase.

## 3. Model Simulation

### 3.1. Parameter Settings

To capture the dominant multiphysics coupling—including fluid dynamics, charge kinetics, and interfacial movement—a 2D cross-sectional model of the electrowetting pixel was established. The primary geometric dimensions and material properties are configured to replicate an authentic experimental environment, as summarized in Table 1.

### 3.2. Governing Equations and Physics Interfaces

The multiphysics simulation model is developed and solved using COMSOL Multiphysics 6.4 within a continuum mechanics framework, integrating laminar flow, phase-field, electrostatics, and global ordinary differential equations (ODEs) to capture the complex dynamics of electrowetting. Both the polar liquid and the oil are treated as incompressible Newtonian fluids, whose motion is governed by the Navier–Stokes equations and the continuity equation, as shown in Equation (1) [32,33].

(1) $$\begin{array}{c} \rho \left(\frac{\partial u}{\partial t}+u\cdot \nabla u\right)=\nabla \cdot \left(\mu \left(\nabla u+{\left(\nabla u\right)}^{T}\right)-pI\right)+F_{st}+F_{vf} \end{array}$$

where ρ is the fluid density, u is the velocity vector, t is the time, p is the static pressure, μ is the dynamic viscosity, and I denotes the identity tensor, which characterizes the isotropic distribution of the pressure field. The source terms $F_{st}$ and $F_{vf}$ represent the surface tension force and the volume force generated by the electrostatic field, respectively.

The topological evolution of the oil–water interface is captured using the phase-field method. This approach introduces a dimensionless order parameter, ϕ (the phase-field variable), where ϕ=−1 represents the oil phase, and ϕ=1 represents the water phase. Within the interface transition zone, ϕ varies continuously between −1 and 1. At the two-phase interface, ρ and μ are determined through linear interpolation based on the phase field variable, as shown in Equations (2) and (3) [34].

(2) $$\begin{array}{c} \rho =\rho_{oil}+(\rho_{water}-\rho_{oil})\frac{1+\phi }{2} \end{array}$$

(3) $$\begin{array}{c} \mu =\mu_{oil}+{(\mu }_{water}-\mu_{oil})\frac{1+\phi }{2} \end{array}$$

where $\rho_{oil}$ and $\rho_{water}$ are the densities of oil and water, respectively; $\mu_{oil}$ and $\mu_{water}$ are the dynamic viscosities of oil and water, respectively. The evolution of the phase interface is governed by the Cahn–Hilliard equation, which is decomposed into two second-order partial differential equations for numerical stability. These processes are described by Equations (4) and (5) [35,36].

(4) $$\begin{array}{c} \frac{\partial \phi }{\partial t}+u\cdot \nabla \phi =\nabla \cdot \frac{\gamma_{0}\lambda }{\varepsilon^{2}}\nabla \psi \end{array}$$

(5) $$\begin{array}{c} \psi =-\nabla \cdot \varepsilon^{2}\nabla \phi +\left(\phi^{2}-1\right)\phi \end{array}$$

where $\gamma_{0}$ is the mobility parameter, λ is the mixing energy parameter, ε is the capillary width varying with the interface thickness, and ψ is the chemical potential. These parameters are related to the interfacial tension coefficient σ and are described by Equations (6) and (7).

(6) $$\begin{array}{c} \lambda =\frac{3\varepsilon \sigma }{\sqrt{8}} \end{array}$$

(7) $$\begin{array}{c} {\gamma_{0}=\chi \varepsilon }^{2} \end{array}$$

where χ is the mobility adjustment parameter. Surface tension is incorporated as an equivalent body force, as shown in Equations (8) and (9).

(8) $$\begin{array}{c} F_{st}=G\nabla \phi \end{array}$$

(9) $$\begin{array}{c} G=\frac{\lambda }{\varepsilon^{2}}\psi \end{array}$$

where G is the chemical potential of the system. The driving force for electrowetting originates from the electrostatic field. Since the bulk fluids contain no free volume charges, the electric potential distribution is governed by Equations (10)–(12) [37,38].

(10) $$\begin{array}{c} \nabla \cdot D=\rho_{v} \end{array}$$

(11) $$\begin{array}{c} E=-\nabla V \end{array}$$

(12) $$\begin{array}{c} D=\varepsilon_{0}\varepsilon_{r}E \end{array}$$

where D is the electric displacement, $\rho_{v}$ is the volume charge density, E is the electric field strength, V is the electric potential, $\varepsilon_{0}$ is the permittivity of vacuum, and $\varepsilon_{r}$ is the relative permittivity. The force exerted by the electric field on the fluid is calculated through the divergence of the MST, as shown in Equations (13) and (14) [39,40].

(13) $$\begin{array}{c} F_{vf}=\nabla \cdot T \end{array}$$

(14) $$\begin{array}{c} T=\varepsilon_{0}\varepsilon_{r}[EE-\frac{1}{2}(E\cdot E)I] \end{array}$$

The tensor T is defined using Equation (14), representing the mechanical stress induced by the electrostatic field within dielectric media. In the absence of free volume charges ($\rho_{v}=0$), the divergence of the MST can be simplified using vector identities and Gauss’s law, as shown in Equation (15).

(15) $$\begin{array}{c} F_{vf}=-\frac{1}{2}(E\cdot E)\nabla \varepsilon_{0}\varepsilon_{r} \end{array}$$

Because ε varies abruptly only at the oil–water interface, the electric force is precisely localized at the boundary, effectively driving the contraction or spreading of the oil.

### 3.3. Dynamic Contact Angle and MKT Coupling

To characterize the physical pinning effect and energy dissipation during the wetting process, the MKT is coupled at the wetted wall boundary of the simulation. Within this macroscopic framework, the intricate non-equilibrium dynamics of the moving contact line described by Equation (16) are conventionally simplified into a friction-based formulation, as expressed in Equation (17).

(16) $$\begin{array}{c} v_{cl}=2\kappa^{0}\lambda_{jump}\sinh(\frac{\gamma_{ow}\lambda_{jump}^{2}}{2k_{B}T}(\cos\theta_{eq}-\cos\theta_{dyn})) \end{array}$$

(17) $$\begin{array}{c} \cos\theta_{dyn}=\cos\theta_{eq}-\zeta_{MKT}\cdot \mathrm{arsinh}\left(\frac{v_{cl}}{V_{0}}\right) \end{array}$$

where $v_{cl}$ represents the velocity of the TCL moving along the wetted wall; $k_{B}$ is the Boltzmann constant; T is the absolute thermodynamic temperature; $\gamma_{ow}$ is the surface tension of the oil–water interface; and $\theta_{eq}$ and $\theta_{dyn}$ denote the static and dynamic contact angles, respectively. $\lambda_{jump}$ and $\kappa^{0}$ serve as the two primary microscopic fitting parameters. Specifically, $\lambda_{jump}$ is directly associated with the microscopic roughness and defect density of the dielectric surface, defining the spatial scale of the physical pinning effect. Meanwhile, $\kappa^{0}$ reflects the microscopic mobility of oil molecules at the TCL. $V_{0}$ represents the characteristic molecular jump velocity. The term $\zeta_{MKT}$ denotes the microscopic friction coefficient reflecting surface roughness and chemical defects, defined as $\zeta_{MKT}=\frac{k_{B}T}{\kappa^{0}\lambda_{jump}^{3}}$. This model accurately captures the “stick-slip” behavior and energy dissipation of the oil during the initiation and termination phases of motion. $\theta_{eq}$ is governed by the Young–Lippmann equation, as shown in Equation (18) [27].

(18) $$\begin{array}{c} \cos\;\theta_{eq}=\cos{\;\theta }_{0}+\frac{\varepsilon_{0}\varepsilon_{d}}{2\gamma_{ow}d}U^{2} \end{array}$$

where $\theta_{0}$ is the initial static contact angle at zero voltage; $\varepsilon_{0}$ is the vacuum permittivity; $\gamma_{ow}$ is the surface tension of the oil–water interface; d and $\varepsilon_{d}$ are the thickness and relative permittivity of the dielectric layer, respectively; and U is the applied voltage.

To implement contact angle hysteresis at the wetted wall boundary and ensure numerical stability, a robust MKT coupling expression is constructed to prevent domain-related errors of the inverse cosine function. During numerical iterations, potential oscillations may cause the computed cosine value to slightly exceed the physical range of [−1, 1], leading to solver failure. Therefore, the dynamic contact angle is formulated as Equation (19).

(19) $$\theta_{dyn}=acos\left[max\left(-0.999,\;min\left(cos\;\theta_{eq}-\zeta_{MKT}\cdot arsinh\left(\frac{v_{cl}}{V_{0}}\right)\right)\right)\right]$$

By utilizing bounding functions to strictly constrain the argument within the [−0.999, 0.999] interval, the model effectively circumvents non-convergence issues caused by numerical fluctuations.

### 3.4. Charge Accumulation Feedback Loop

In addition to contact angle hysteresis, another pivotal factor contributing to the degradation of pixel performance is the charge-trapping effect within the dielectric layer. To simulate the shielding effect of trapped charges on the effective driving voltage, a global ODE is introduced to quantify the dynamic evolution of the trapped charge density. Under the influence of an external electric field, charge carriers are injected into the dielectric bulk. According to the phenomenological kinetic model, the temporal evolution of the trapped charge is formulated as Equation (20).

(20) $$\begin{array}{c} \frac{dQ_{trap}}{dt}=k_{inj}V_{app}(t)-\frac{Q_{trap}}{\tau } \end{array}$$

where $Q_{trap}$ is the temporal evolution of the trapped charge, $k_{inj}$ is the charge injection efficiency constant characterizing the rate of carrier injection, $V_{app}(t)$ is the time-dependent externally applied driving waveform, and τ is the characteristic decay time constant representing the relaxation rate of the trapped charges. These accumulated charges establish an internal reverse electric field that partially shields the external field. Consequently, the effective driving voltage is corrected using Equation (21).

(21) $$\begin{array}{c} V_{eff}=V_{app}(t)-\alpha Q_{trap} \end{array}$$

where $V_{eff}$ is the effective driving voltage, and α is the electrostatic shielding coefficient, which quantifies the reduction in potential per unit of trapped charge. In this paper, the Global ODEs interface in COMSOL Multiphysics is utilized to solve for $Q_{trap}$ in real time, and the resulting values are dynamically fed back into the terminal boundary condition of the electrostatics interface. Through this feedback loop, the simulation model accurately captures nonlinear phenomena such as voltage saturation, grayscale drift, and sluggish response times induced by charge accumulation.

### 3.5. Mesh Refinement and Simulation Coupling

Considering the sharp local potential gradients induced by charge trapping and the extreme velocity gradients near the three-phase contact line, a non-uniform meshing strategy is employed to balance computational efficiency and numerical accuracy. High-density boundary layers and mapped meshes are implemented at the wetted wall boundaries and within the anticipated regions of oil–water interface evolution. To accurately capture the microscopic slippage of the contact line and the shielding effects resulting from charge accumulation, the maximum element size, denoted as $h_{max}$, is strictly controlled within 1.0 μm.

Furthermore, the mesh aspect ratio, defined as the ratio of the longest side to the shortest side of an element, is optimized near the interfaces to suppress numerical diffusion during rapid transitions of ϕ. This refined discretization scheme ensures that the model precisely resolves the electric pressure gradients at the dielectric surface and effectively couples the microscopic friction forces defined by the MKT. By implementing localized refinement in critical physical regions, the simulation not only eliminates numerical oscillations of the inverse cosine function during contact angle calculations but also provides a high-fidelity background field for solving $Q_{trap}$ via the Global ODEs.

To ensure the spatial convergence of the numerical results, a mesh independence study was performed by varying the minimum element size from 1.0 μm to 0.25 μm. The deviation in the steady-state aperture ratio was found to be less than 5% when the mesh was refined beyond 0.5 μm. Consequently, a maximum element size of 0.5 μm at the interface was selected to balance computational cost and numerical fidelity.

**Figure 2:**
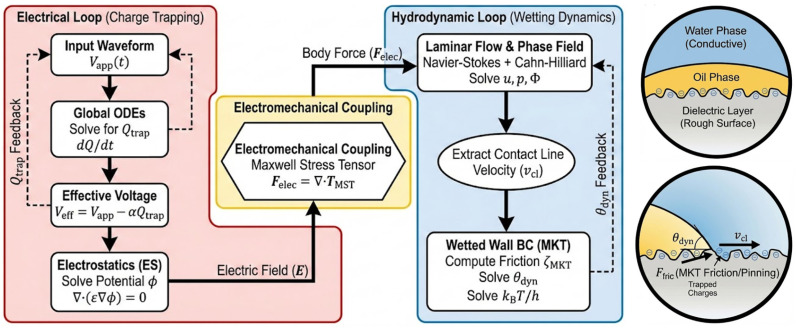
Schematic diagram of the numerical implementation and multiphysics coupling strategy. The simulation framework integrates three primary modules: the phase-field method coupled with laminar flow to track the topological evolution of the oil–water interface driven by the Maxwell stress tensor and surface tension; the MKT mechanism implemented at the wetted wall boundary to dynamically update the contact angle based on the contact line velocity; and the electrostatics interface coupled with Global ODEs to solve for the effective potential distribution under charge trapping.

Based on the aforementioned module configurations, $V_{eff}$ is coupled with the MKT to derive the governing equation for the dynamic contact angle, as shown in Equation (22). By substituting $V_{eff}$ into the modified Young–Lippmann framework, the instantaneous equilibrium state of the TCL is dynamically updated. This integration ensures that the simulated oil motion accounts for the synergistic effects of both electrical shielding and hydrodynamic resistance. The coupling flowchart is shown in Figure 2.

(22) $$\begin{array}{c} \theta_{dyn}=acos\left[max\left(-0.999,\;min\left(cos{\;\theta }_{0}+\frac{\varepsilon_{0}\varepsilon_{d}}{2\gamma_{ow}d}{\left(V_{app}(t)-\alpha Q_{trap}\right)}^{2}-\zeta_{MKT}\cdot arsinh\left(\frac{v_{cl}}{V_{0}}\right)\right)\right)\right] \end{array}$$

## 4. Simulation Results and Discussion

### 4.1. Model Validation and Benchmarking

In the 2D simulation model, the AR is characterized by the normalized length of the exposed substrate region. Numerically, this is implemented by performing a line integral of the phase-field distribution function along the bottom boundary representing the dielectric surface, as formulated in Equations (23) and (24).

(23) $$\begin{array}{c} AR=\frac{1}{L}\int_{\Gamma_{bottom}}\;f(\phi )dl \end{array}$$

(24) $$\begin{array}{c} f(\phi )=\frac{1+\phi }{2} \end{array}$$

where $\Gamma_{bottom}$ is the geometric boundary line at the pixel bottom, L is the total width of the pixel, and dl is the differential line element along the boundary. The term f(ϕ) acts as a normalized interpolation function of ϕ to distinguish between the fluid phases: it yields a value of 1 when the point is fully occupied by the aqueous phase (ϕ=1) and 0 when covered by the oil (ϕ=−1). This integral quantifies the degree of oil contraction and the corresponding optical modulation performance by calculating the fractional length of the substrate in contact with the polar liquid under the applied electric field.

**Figure 3:**
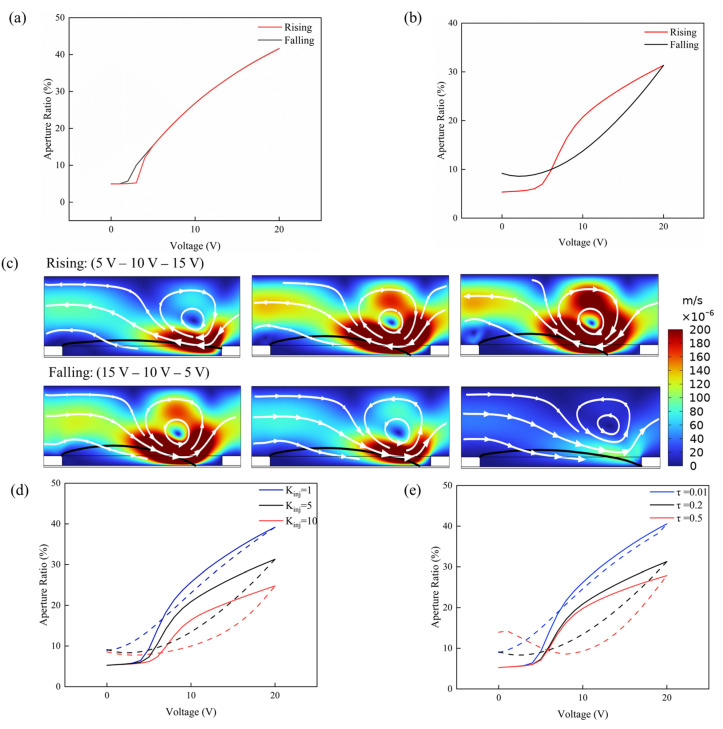
(**a**) AR-V response curves under the idealized limit. (**b**) AR-V response curves under the coupled module. (**c**) EHD-driven flow field and interfacial evolution. The black contour indicates the oil–water interface. The background color and superimposed white streamlines represent the velocity magnitude and flow direction, respectively. The results clearly reveal a distinct recirculation vortex induced by concentrated Maxwell stress near the contact line. (**d**) AR-V response curves under different charge injection efficiency constants. The solid line represents the voltage rising phase, and the dashed line represents the voltage falling phase. (**e**) AR-V response curves under different characteristic charge decay time constants. The solid line represents the voltage rising phase, and the dashed line represents the voltage falling phase.

Under the idealized limit (setting the charge injection constant $k_{inj}=0$ and neglecting charge-trapping effects), the AR of the system was evaluated across a range of swept driving voltages to verify its adherence to the theoretical Young–Lippmann curve. This benchmark test was designed to establish the fundamental accuracy of the multiphysics coupling between the electrostatic and phase-field interfaces in the absence of electrical defects. As illustrated in Figure 3a, the simulated AR-V curve agrees well with the theoretical prediction and exhibits nearly coincident rising and falling paths, verifying the reliability of the model in describing interfacial evolution and contact line equilibrium.

The initial AR exhibits a non-zero baseline value of approximately 5% to 8% under zero-voltage conditions. This arises because the phase-field method introduces a diffused interfacial layer with a finite capillary width. Since the interfacial layer numerically spans several mesh elements, the interfacial transition zone is partially identified as the opened region during the integration process. This numerically induced baseline value has no impact on the subsequent quantitative analysis of the dynamic hysteresis loops.

By incorporating charge-trapping kinetics, the electrostatic screening effect arising from the gradual accumulation of interfacial charges is simulated, as shown in Figure 3b,c. Furthermore, the effects of the charge injection efficiency constant $k_{inj}$ and time constant *τ* on the hysteresis curve are quantitatively investigated.

Figure 3d illustrates the AR versus voltage hysteresis loops under varying charge injection efficiency constants ($k_{inj}$ = 1, 5, and 10 S). It is clearly observed that the charge injection efficiency constant significantly dictates both the maximum achievable AR and the area of the hysteresis loop. During the voltage rising phase, as the charge injection efficiency constant increases from 1 to 10 S, the maximum AR at 20 V drops substantially from approximately 40% to 22%. This degradation occurs because a higher injection efficiency constant accelerates the accumulation of trapped charges at the dielectric interface. These trapped carriers establish an internal remnant electric field that opposes the externally applied voltage, thereby inducing a strong electrostatic screening effect that diminishes the effective Maxwell stress driving the oil contraction. During the voltage-falling phase, the system with the highest injection efficiency constant (rate = 10 S) exhibits a severely depressed falling curve, resulting in a pronounced widening of the hysteresis loop. This indicates that the heavily accumulated interfacial charges cannot completely undergo relaxation or dissipation in a short time. Consequently, the effective voltage acting on the TCL is significantly lower than the externally applied voltage during ramp-down, which exacerbates the dynamic energy dissipation and broadens the contact angle hysteresis.

Figure 3e illustrates the AR versus voltage hysteresis loops under different characteristic charge decay time constants (*τ* = 0.01, 0.2, and 0.5 s). It is clearly evident that the charge relaxation rate plays a decisive role in both the steady-state response and the degree of hysteresis. During the voltage rising phase, when the decay time *τ* is short (*τ* = 0.01 s), the charges accumulated at the interface can dissipate rapidly. The system experiences a weaker electrostatic screening effect, thereby reaching a higher maximum AR of approximately 40% at 20 V. As *τ* increases to 0.5 s, the extremely slow charge dissipation leads to the formation of a strong remnant built-in electric field. This significantly attenuates the effective driving voltage, causing the maximum AR to drop to roughly 25%. During the voltage-falling phase, a longer decay time severely exacerbates the CAH. For the condition of *τ* = 0.5 s, the trapped charges require a significantly longer time to be released as the external voltage is reduced. Consequently, the receding curve deviates drastically from the advancing curve, resulting in an exceptionally broad hysteresis loop. Conversely, when *τ* = 0.01 s, the rapid charge relaxation allows the receding path to closely track the advancing path, effectively mitigating the hysteresis effect. This confirms the critical role of charge relaxation in determining the dynamic equilibrium and provides a physical basis for analyzing the oil contraction limits under high-voltage driving.

### 4.2. Influence of Driving Waveforms on the Hysteresis Effect

**Figure 4:**
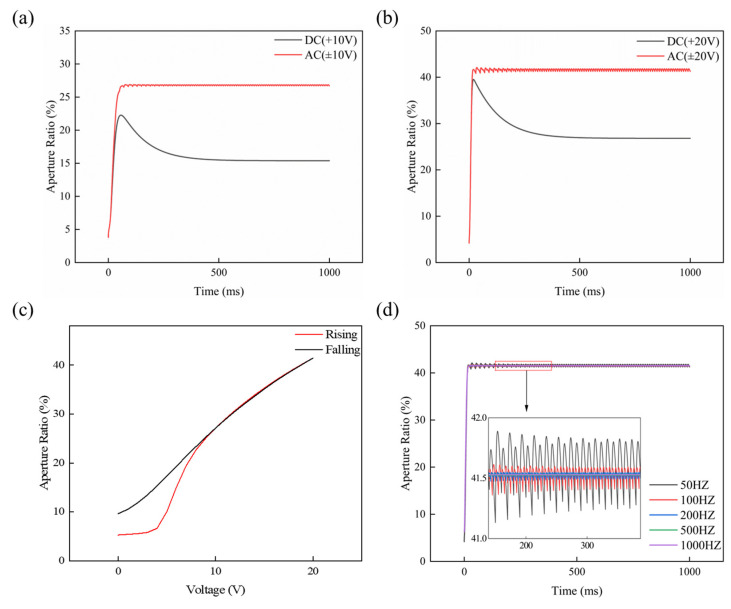
(**a**) AR-T response curves under 10 V DC and AC waveform driving. (**b**) AR-T response curves under 20 V DC and AC waveform driving. (**c**) AR-V response curves under AC waveform driving. (**d**) AR-T response curves at different frequencies under 20 V DC waveform driving.

Under DC waveform driving, trapped charges on the dielectric surface accumulate unidirectionally, causing a gradual decrease in the AR. By contrast, AC waveform driving uses periodic polarity reversal to force carriers injected into the dielectric layer to recombine or migrate in the reverse direction within half a cycle, as shown in Figure 4a,b. This dynamic equilibrium keeps residual charge at an extremely low level, thus inhibiting the formation of a built-in screening field and ensuring a consistent, effective driving force during both voltage ramp-up and ramp-down phases.

As illustrated in Figure 4c, the AC waveform driving significantly suppresses contact angle hysteresis, resulting in a near-perfect overlap of the rising and falling curves above 5 V. This high reversibility is primarily attributed to the periodic polarity reversal, which effectively mitigates interfacial charge trapping and prevents the formation of an internal electrostatic screening field. Simulation results indicate that the hysteresis loop area first decreases and then plateaus with increasing frequency. At low frequencies, the oil–water interface has sufficient time to respond to voltage polarity switching, leading to visually perceptible periodic fluctuations in the AR (the low-frequency oscillation effect). When the frequency rises above 500 Hz, interfacial displacement is jointly constrained by μ and $\zeta_{MKT}$, making it unable to track the variation of the transient electric field. Consequently, the interface enters a quasi-steady oscillation state, which macroscopically presents a stable AR. This characteristic is referred to as the high-frequency quasi-steady-state effect, as shown in Figure 4d.

**Figure 5:**
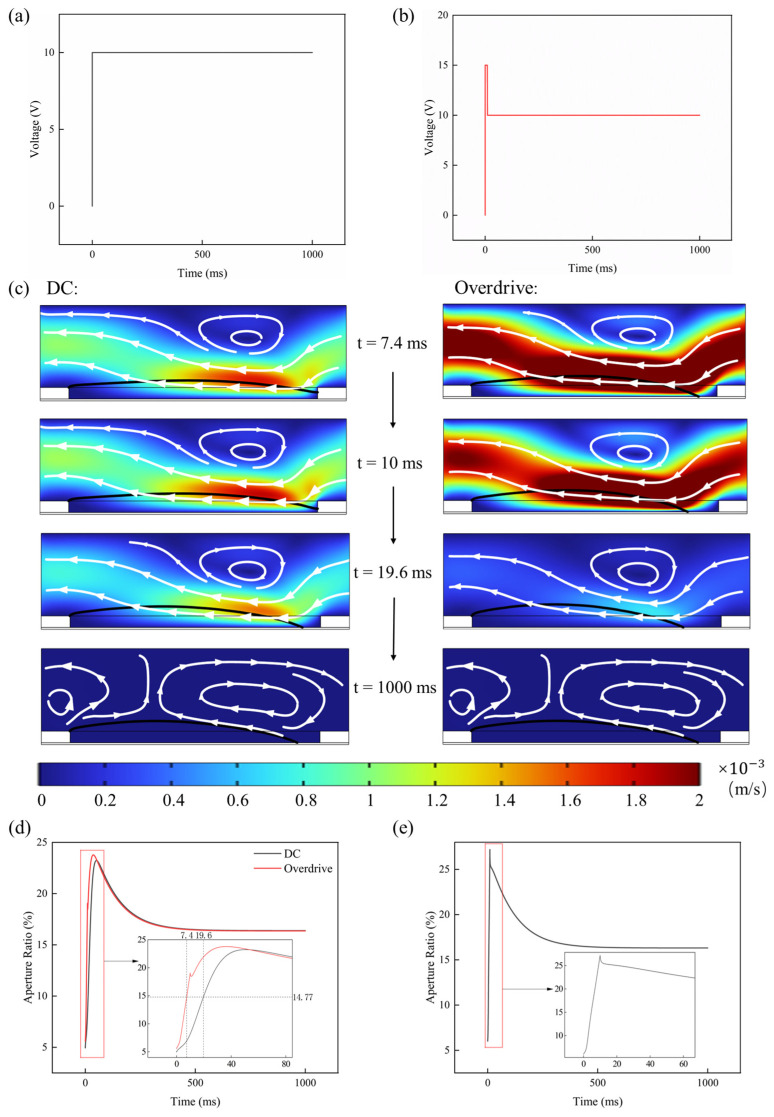
(**a**) Schematic diagram of the 10 V DC waveform driving. (**b**) Schematic diagram of the overdrive waveform ($V_{OD}=15\;V$ for a duration of $t_{OD}=10\;ms$, followed by $V_{target}=10\;V$) driving. (**c**) Comparison of simulated oil states at different times under DC and overdrive waveform driving. (**d**) Comparison of AR under DC and overdrive waveform driving. (**e**) Phenomenon of overdrive overshoot.

In practical applications, initiating interfacial motion requires overcoming a dual energy barrier: a static thermodynamic barrier imposed by surface heterogeneity and an electrostatic potential barrier induced by trapped charges. To alleviate the prolonged response time caused by conventional step-voltage driving, an overdrive strategy is implemented [15]. The overdrive waveform consists of a suprathreshold transient voltage pulse ($V_{OD}$) applied during the rising phase, which is considerably higher than the target holding voltage ($V_{target}$) and lasts for a short duration ($t_{OD}$), as shown in Figure 5a,b.

Figure 5c,d present a comparative analysis of the AR evolution over time under standard DC waveform driving ($V_{target}=10\;V$) and overdrive driving ($V_{OD}=15V$ for a duration of $t_{OD}=10\;ms$, followed by $V_{target}=10\;V$). Due to significant viscous dissipation and gradual charge screening, the standard driving scheme requires approximately 19.6 ms to reach 90% of the steady-state AR. In sharp contrast, the overdrive strategy generates a high initial interfacial velocity, enabling the system to attain the target state within 7.4 ms, thereby substantially accelerating the dynamic response. This enhanced dynamic response is fundamentally attributed to two mechanisms confirmed by the simulation: first, the high-amplitude $V_{OD}$ pulse generates a large transient electrostatic pressure gradient that provides sufficient mechanical work to immediately overcome the local static friction threshold defined by the MKT model; second, the short $t_{OD}$ circumvents the electrostatic screening effect during the critical acceleration phase, as it accelerates the interface before significant charge accumulation occurs.

Notably, optimization of pulse parameters ($V_{OD}$ and ${\;t}_{OD}$) is crucial. Excessively high overdrive voltage or prolonged duration leads to inertial overshoot and subsequent underdamped oscillations of the interface around the equilibrium position, which is detrimental to optical stability, as shown in Figure 5e. The proposed multiphysics framework provides a quantitative tool to determine the required optimal energy injection, balancing rapid depinning with viscous damping to ensure a fast response with critical damping.

## 5. Conclusions

This paper establishes a high-fidelity EHD simulation framework that rigorously couples the Cahn–Hilliard phase-field equation, the MKT, and charge transport kinetics in the dielectric layer. This framework provides a quantitative tool for elucidating the non-equilibrium dynamics of droplet manipulation in micro-confined geometries. Building upon this framework, we successfully achieve the quantitative decoupling of the energy dissipation mechanisms controlling the moving contact line. Specifically, the numerical analysis reveals that while hydrodynamic friction sets the baseline resistance, interfacial charge accumulation is the dominant factor exacerbating contact angle hysteresis and residual displacement. Furthermore, comparative analysis of flow dynamics confirms that the AC waveform driving significantly alters the interfacial stress distribution, inducing micro-oscillations that lower the activation energy barrier for depinning. Conversely, the overdrive strategy utilizes a transient suprathreshold Maxwell stress to overcome initial viscous damping and achieve a critically damped response. These findings offer a solid theoretical foundation for understanding the stability of confined droplets and provide general scaling laws for optimizing active driving waveforms in next-generation digital microfluidics and optofluidic devices.

Despite these insights, the current 2D cross-sectional model is limited by its assumption of an infinite trench geometry, which inherently neglects the 3D azimuthal curvature of the droplet and localized pinning effects at the pixel corners. Furthermore, while the macroscopic friction coefficient accounts for generalized surface roughness, it cannot fully capture stochastic manufacturing variations, such as localized porosity in the hydrophobic coating or dielectric thickness fluctuations. Future work will focus on developing a fully coupled 3D simulation framework and integrating it with empirical experiments to investigate the impact of actual fabrication defects on dynamic droplet behavior.

## Figures and Tables

**Table 1:** Electrowetting pixel manufacturing process parameters.

Parameters	Quantity	Value	Unit
Material	Density of oil	735	kg/m^3^
	Density of water	999.62	kg/m^3^
	Dynamic viscosity of oil	0.941	mPa·s
	Dynamic viscosity of water	1.01	mPa·s
	Relative dielectric constant of oil	2.2	1
	Relative dielectric constant of water	80	1
	Relative dielectric constant of hydrophobic insulating layer	1.934	1
	Relative dielectric constant of pixel wall	3.28	1
	Microscopic friction coefficient	0.5	/
Structure	Width of pixel	160	μm
	Width of pixel wall	15	μm
	Height of pixel wall	3.5	μm
	Thickness of hydrophobic insulating layer	0.5	μm
	Thickness of oil	3.5	μm
Interfacial	Surface tension of oil and water	0.02	N/m
	Contact angle at the top of the pixel wall	70	deg
	Contact angle on the side of the pixel wall	90	deg
	Contact angle of hydrophobic insulating layer	165	deg
	Jump velocity of characteristic molecular motion	0.001	m/s
	Charge injection efficiency constant	5	S
	Characteristic decay time constant	0.2	s

## Data Availability

All data that support the findings are included in the manuscript.

## Abbreviations

The following abbreviations are used in this manuscript:

CAH	Contact Angle Hysteresis
EHD	Electrohydrodynamic
MKT	Molecular Kinetic Theory
TCL	Three-Phase Contact Line
AC	Alternating Current
DC	Direct Current
AR	Aperture Ratio
ODEs	Ordinary Differential Equations
MST	Maxwell Stress Tensor
